# Modulation of RNA primer formation by Mn(II)-substituted T7 DNA primase

**DOI:** 10.1038/s41598-017-05534-3

**Published:** 2017-07-19

**Authors:** Stefan Ilic, Sabine R. Akabayov, Roy Froimovici, Ron Meiry, Dan Vilenchik, Alfredo Hernandez, Haribabu Arthanari, Barak Akabayov

**Affiliations:** 1 Department of Chemistry, Ben-Gurion University of the Negev, Beer-Sheva, 8410501 Israel; 2 Department of Communication Systems Engineering, Ben-Gurion University of the Negev, Beer-Sheva, 8410501 Israel; 3 Department of Biological Chemistry and Molecular Pharmacology, Harvard Medical School, 240 Longwood Ave., Boston, Massachusetts 02115 USA

## Abstract

Lagging strand DNA synthesis by DNA polymerase requires RNA primers produced by DNA primase. The N-terminal primase domain of the gene 4 protein of phage T7 comprises a zinc-binding domain that recognizes a specific DNA sequence and an RNA polymerase domain that catalyzes RNA polymerization. Based on its crystal structure, the RNA polymerase domain contains two Mg(II) ions. Mn(II) substitution leads to elevated RNA primer synthesis by T7 DNA primase. NMR analysis revealed that upon binding Mn(II), T7 DNA primase undergoes conformational changes near the metal cofactor binding site that are not observed when the enzyme binds Mg(II). A machine-learning algorithm called linear discriminant analysis (LDA) was trained by using the large collection of Mn(II) and Mg(II) binding sites available in the protein data bank (PDB). Application of the model to DNA primase revealed a preference in the enzyme’s second metal binding site for Mn(II) over Mg(II), suggesting that T7 DNA primase activity modulation when bound to Mn(II) is based on structural changes in the enzyme.

## Introduction

In about a third of all enzymes, biological function is dependent on the presence of a divalent metal cofactor in the enzyme’s active site^1^. Metal ions play essential roles in the active sites of enzymes by stabilizing the transition state of the enzyme-catalyzed reaction and by orienting the substrate for optimal binding with the enzyme^2^. The formation and stability of the metal-enzyme complex depend on the properties of both the metal (valence state, ionic radius, charge-accepting ability) and the amino acids that constitute the metal binding site (net charge, dipole moment and polarizability, electron donor and acceptor ability, etc.)^3^. The effects of these properties manifest in the metal ion’s coordination number which, in conjunction with its coordination geometry (arrangement of the ligands around the metal in 3D space), determines the structure and overall properties of the enzyme-metal complexes^4^. The metal binding sites of many enzymes are capable of binding more than one type of metal^5–7^. For example, owing to their similarity in size and charge, the metal ions Mg(II) and Mn(II), the subjects of our study, can substitute for each other in a large variety of enzymes^8^.

Experiments with Mg(II)-dependent enzymes showed that enzyme catalytic activity is often maintained when Mn(II) is substituted as the metal cofactor^8^. Although the two metal ions share preferences for a coordination number of six and a regular octahedral arrangement^2, 9^, Mg(II) usually binds oxygen (typically from water molecules) while Mn(II) has a marginally higher affinity for nitrogen^8^ and a tendency to bind fewer than six water molecules^2^. Regardless of these differences, Mn(II) can often replace Mg(II) in nucleic acid processing enzymes. However, tests of the reverse case, the substitution of Mn(II) by Mg(II), showed that Mg(II) is less often a catalytically competent replacement for Mn(II) in enzymes^10^. This outcome is likely the result of, among other parameters, structural variations between the metal binding sites of the enzymes.

Insofar as the Mg(II) ion is invisible to most spectroscopic methods, including NMR, structural studies of the metal binding sites in enzymes must exploit methodologies such as x-ray crystallography and x-ray absorption spectroscopy that are amenable to the substitution of Mg(II) with a “visible” metal ion^10–12^. Although NMR is not commonly used to perform structural studies of enzyme metal binding sites, it can provide a fingerprint of the active site residues when Mn(II) or Mg(II) is bound. NMR can also inform us as to whether structural changes occur when Mn(II) is substituted by Mg(II)^13, 14^.

**Figure 1:**
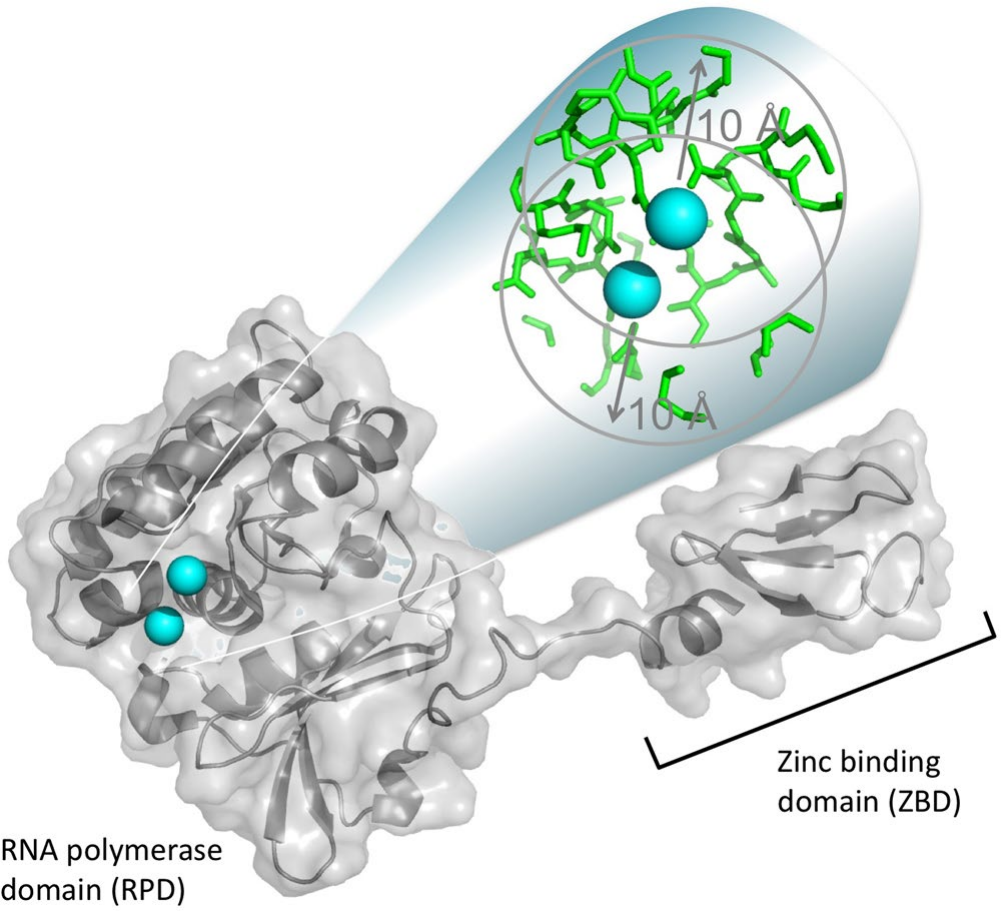
Crystal structure of the phage T7 DNA primase domain of the gene 4 product (PDB entry 1 nui). The two-metal cofactor binding site is emphasized. The residues located within a radius of 10 Å of Mg(II) are colored green. The figure was created using PyMOL (http://www.pymol.org).

At its physiological concentration (i.e., millimolar-range), Mn(II) alters the activities of a variety of DNA and RNA processing enzymes^15, 16^, including that of the DNA primase of bacteriophage T7, the enzyme used in this study. T7 primase is located in the N-terminal half of the multifunctional gene 4 helicase-primase and synthesizes the oligoribonucleotide primers needed for lagging strand DNA synthesis by replicative DNA polymerases^17^. The primase domain comprises RNA polymerase and zinc-binding domains (Fig. 1), the latter of which is essential for the recognition of a specific template sequence, 5′-GTC-3′, at which the primase synthesizes primers^17^. This enzyme was shown to have a conserved domain, known as the Toprim fold^18^, which consists of a glutamate residue and an aspartate-x-aspartate (DxD) motif that are positioned proximal to each other to form a triad motif that binds divalent metal ions^19^.

A crystallographic study of the T7 primase revealed that its active site contains two metal ion cofactors located 7.5 Å from each other^20^ (Fig. 1). This observation, taken together with the findings from other protein crystal studies that revealed the number of metal ions present in the active sites of crystallized enzymes^21–24^ and the valuable knowledge gained from kinetic studies of DNA synthesis^25^, led to the proposal of a widely accepted mechanism of catalysis for DNA-processing enzymes whose active sites use two metal ions. Compared to the single-ion mechanism, the recruitment of two metal ions in catalytic processes entails a number of distinct advantages^26–28^, including decreases in certain activation barriers, better accommodation of substrates, and the formation of energetically favorable transition states. In addition, the presence of a second metal ion can decrease the strength of the bridging-ligand field potential, thereby enhancing enzyme binding affinity for other ligands, especially water^26^.

The role of metal ions in the activities of other DNA-processing enzymes can be modeled based on the topoisomerases^18, 28^. DNA-processing enzymes exploit magnesium both for their catalytic activities^29^ and for the structural changes that occur during active site assembly and disassembly. In the topoisomerases, such as DNA gyrase, Mg(II) ions close to the trans-phosphorylation site of the enzyme form ionic networks that connect the anionic centers of the Toprim fold with that in the nucleic acid^30^. According to the two-ion model^31–34^, it is possible that these two metal ions, rather than being fixed in space, undergo coordination rearrangements such that one is constitutively associated with the enzyme while the other is recruited by the nucleic acid^30^.

In many DNA processing enzymes, the cofactor Mg(II) can be replaced with Mn(II). Although it is not the preferred cofactor, Mn(II) has been shown to stimulate the primase activity of the p49 catalytic subunit of human DNA primase by enhancing nucleoside tri-phosphate (NTP) binding and utilization during both initiation and elongation^35^. However, whether this effect is accompanied by structural rearrangements in the active site is not known. When it is the only metal present, Mn(II) interacts with the α-phosphate of the incoming NTP. This scenario implies that the p49 catalytic subunit must contain at least one Mn(II) binding site^35^.

The divalent metal cofactors in the T7 DNA primase are buried and surrounded by an elaborate network of hydrogen bonds provided by a second coordination layer^20^. This arrangement is similar to that of the metal cofactors that function in the catalytically active sites of general ATPases^36, 37^. In this article, we investigate the effect of Mn(II) substitution on the activity of primase and on the local structure at the active site. We also discuss the structural determinants that govern enzyme selectivity for Mn(II) over Mg(II).

## Results

The principles that govern the binding of a metal cofactor to an enzyme’s active site are not fully known, and predictions of metal binding to an active site based on physicochemical properties are usually not accurate. Using a combination of biochemical and biophysical analyses, we were able to explain the basis for elevated primer synthesis activity by Mn(II)-substituted T7 DNA primase. We also characterized the metal binding site of DNA primase when bound to Mg(II) and when bound to Mn(II) based on large-scale structural classification of metal-binding proteins from the PDB.

### Template-Directed Oligoribonucleotide Synthesis

**Figure 2:**
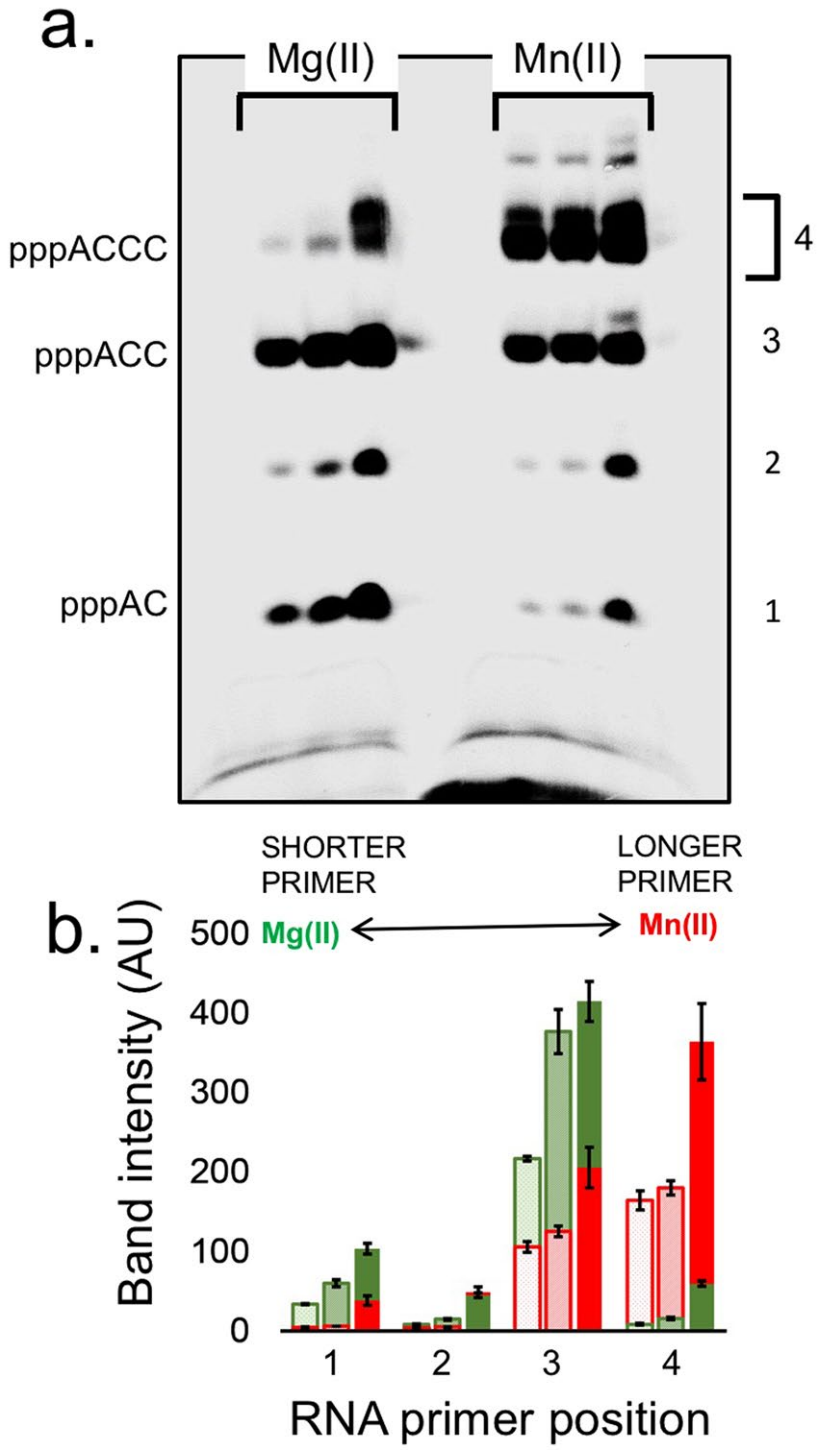
Template-directed oligoribonucleotide synthesis catalyzed by T7 DNA primase. (**a**) The reaction contained the oligonucleotide 5′-GGGTCA_10_-3′ with the primase recognition sequence and [α-^33^P]-CTP and ATP in the standard reaction mixture in the presence of 8 mM MgCl_2_ or 8 mM MnCl_2_ in a reaction buffer containing increasing amounts of T7 DNA primase (0, 1.11, 3.33, and 10 µM). After incubation for 20 min at RT, the radioactive products were analyzed by electrophoresis through a 25% polyacrylamide gel containing 7 M urea and visualized using autoradiography. (**b**) Quantification of primase activity. The bands in the gel presented in a were analyzed using autoradiography.

T7 DNA primase catalyzes the syntheses of the di-, tri-, and tetraribonucleotides pppAC, pppACC, and pppACCC, respectively, on a DNA template containing the T7 primase-recognition site 5′-GGGTC-3′^38^. To determine the effect of substituting Mg(II) with Mn(II), a reaction mixture containing the oligonucleotide 5′-GGGTCA_10_-3′ with the primase recognition sequence, [α-^33^P]-CTP, ATP, and MgCl_2_ or MnCl_2_ was prepared. The radioactively labeled oligoribonucleotide products were separated on a denaturing polyacrylamide gel, and the radioactivity was measured on an autoradiogram. The substitution of Mg(II) with Mn(II) in the reaction buffer resulted in a significant elevation in RNA product formation (Fig. 2).

### Rate of RNA dinucleotide formation in the presence of Mn(II)

**Figure 3:**
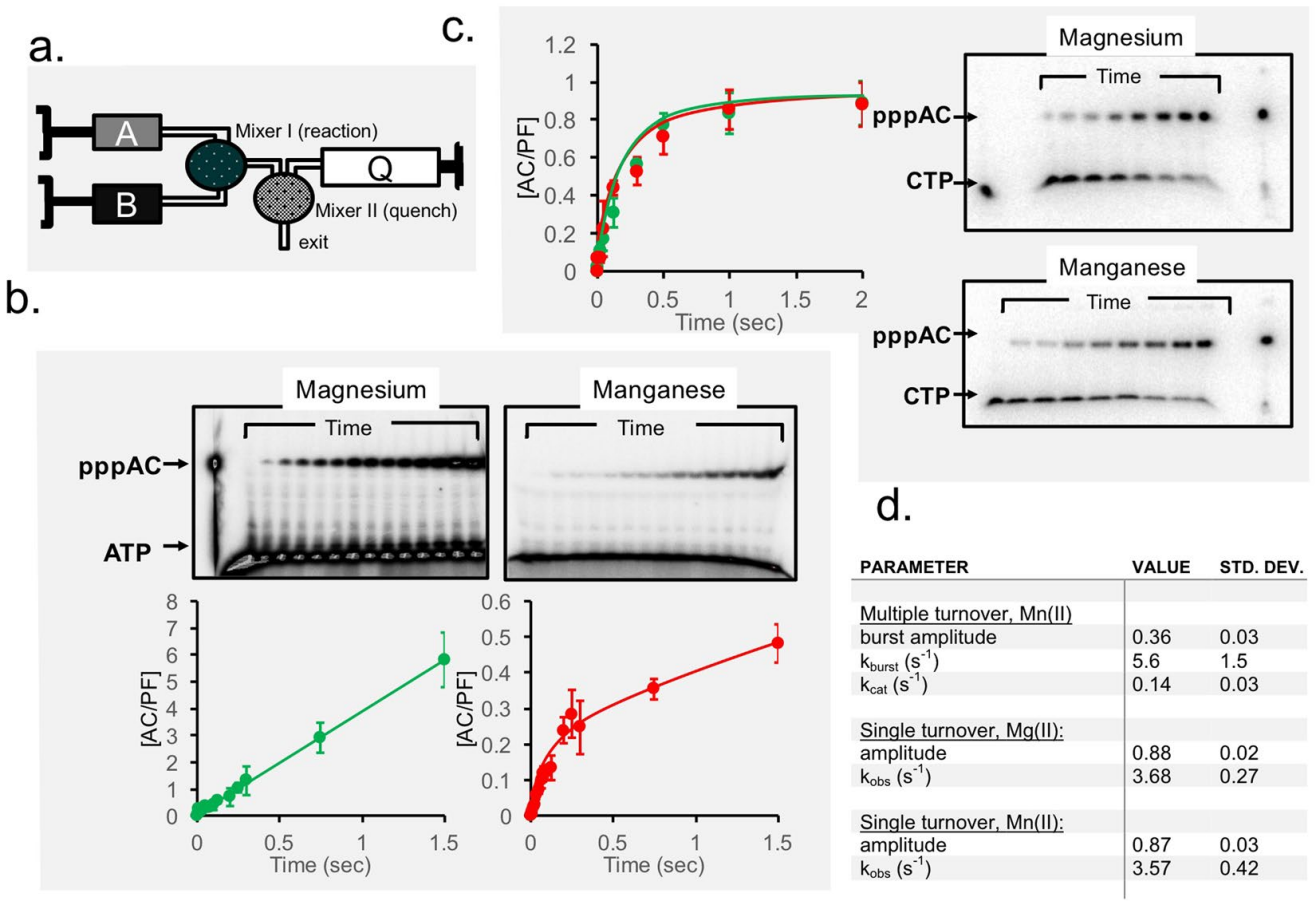
Dinucleotide synthesis by the T7 primase fragment catalyzed by Mn(II) or by Mg(II). (**a**) Experimental setup. (**b**) Single-turnover dinucleotide synthesis was initiated by mixing 25 µM T7 primase and 100 µM template (5′-GTCA_10_-3′) with 5 mM MnCl_2_, 1 nM **γ**-^32^P ATP and 1 mM CTP in a rapid quench-flow instrument. Reactions were quenched after various incubation times, and products were separated by gel electrophoresis. The fraction of dinucleotide product formed as a function of time was plotted for Mg(II) (green curve) and Mn(II) (red curve), and the curves were fit to a single-exponential function. (**c**) Dinucleotide synthesis was initiated by mixing (final concentrations) 10 µM T7 primase and 100 µM template with 10 mM MgCl_2_ or 2 mM MnCl_2_, 1 mM ATP and 1 mM CTP (spiked with [α-^32^P CTP]) in a rapid quench-flow instrument. Reactions were quenched after various incubation times and products were separated by gel electrophoresis. The concentration of dinucleotide formed divided by the enzyme concentration is plotted versus time for MgCl_2_ and MnCl_2_, and the curves were fit to either a linear or a burst equation. (**d**) Table listing the rate constants and the corresponding uncertainties that were used to fit the data.

We explored the effects of the divalent cation Mg(II) or Mn(II) on the kinetics of diribonucleotide synthesis by the T7 primase (setup is shown in Fig. 3a). As shown in Fig. 3b, when the primase was fully saturated with nucleotide substrates, i.e., under *V*_max_ conditions, there was a striking difference in the rate of product accumulation that depended on the identity of the metal cation. In multiple turnover reactions with Mg(II) as the divalent metal, the diribonucleotide product, pppAC, accumulated linearly as a function of time, even in the millisecond time range, displaying a rate constant of ~4 s^−1^ that is consistent with the findings of previous studies^39^. The use of Mn(II) as the cofactor resulted in a biphasic reaction in which AC accumulation exhibited a characteristic pre-steady-state burst of formation. After the initial rapid formation of AC product at a rate constant consistent with what we observed for dinucleotide synthesis in the presence of Mg(II), reaction progress was retarded by a slow steady-state rate once AC was formed. This slower step not only limits the extent to which additional catalytic cycles will restart in the steady state, it also decreases the overall reaction rate.

We also tested whether the rate of the chemical step was dependent on the identity of the divalent metal cofactor by following the kinetics of the single-turnover conversion of ATP to AC in the presence of either MgCl_2_ or MnCl_2_. These experiments indicated that the rates of ATP and CTP condensation are independent of the identity of the metal cofactor, which had no effect on the rates of diribonucleotide formation (Fig. 3c).

These results indicate that the identity of the divalent metal triggered a regulatory transition in the primase catalytic cycle. Thus, in reactions that proceeded in the presence of Mg(II), the rate-limiting step(s) occurred prior to or at AC formation, whereas in the presence of Mn(II), the initial nucleotide condensation reaction was rapid, and a subsequent step, such as primer release, became the limiting step.

### Structural analysis of T7 DNA primase bound to Mn(II)

**Figure 4:**
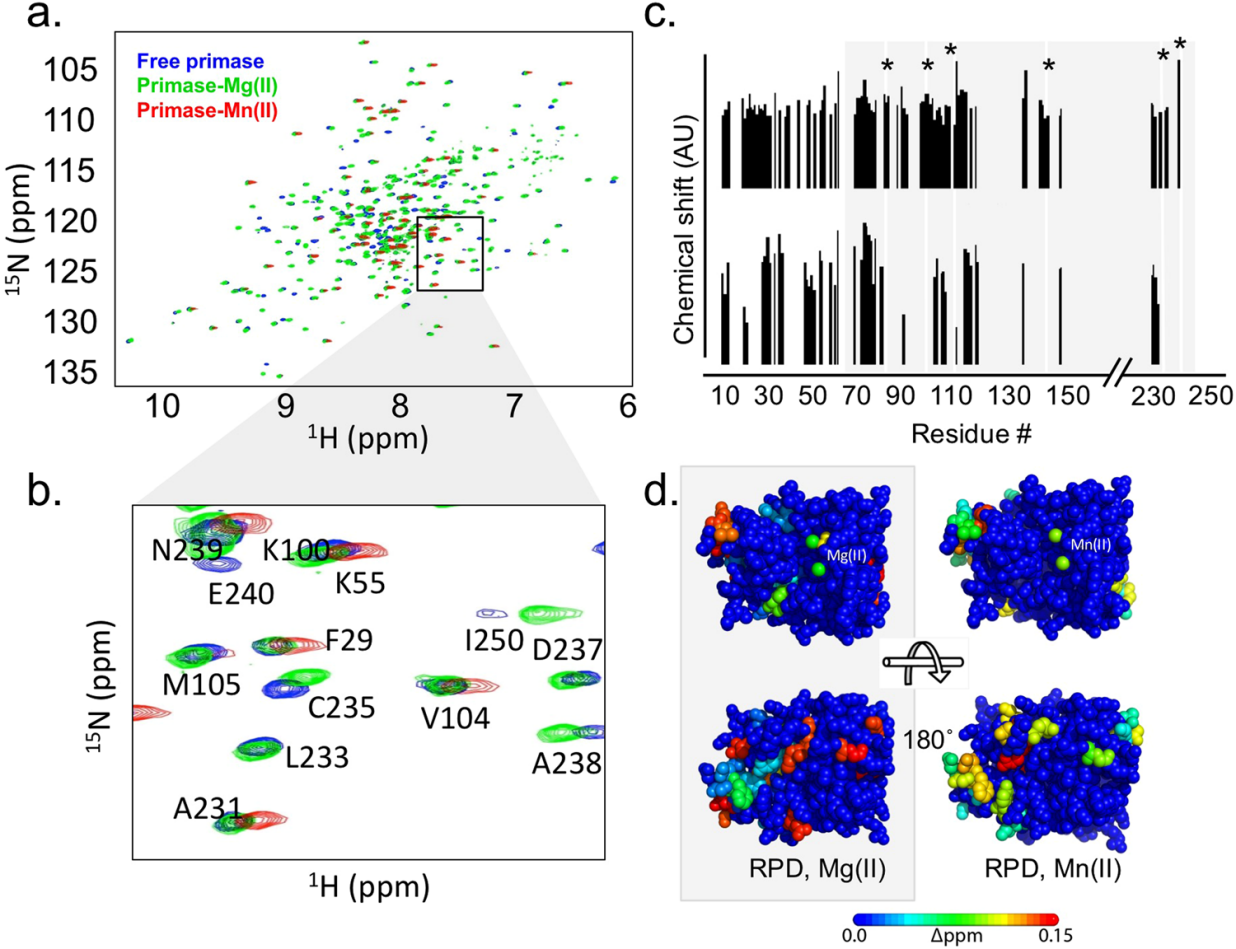
Effect of divalent metal binding on the structure of T7 primase. (**a**) [^1^H,^15^N]-TROSY HSQC titration spectra of ^15^N-deuterated T7 DNA primase without or in the presence of 10 mM Mg(II) or 1 mM Mn(II). NMR experiments for backbone assignments were described recently^40^. Presented are HSQC data for free DNA primase (blue) and for primase bound to Mg(II) (green) and to Mn(II) (red). (**b**) Selected peaks are shown with assigned amino acid resonances as indicated. (**c**) Values of chemical shifts are indicated for Mg(II) (top) and Mn-substituted (bottom) T7 primase. (**d**) RNA polymerase domain (RPD) of T7 primase is indicated in grey box. Residues found 15 Å or closer to a metal ion in the crystal structure are marked with an asterisk.

To characterize the structure of T7 DNA primase bound to either Mg(II) or Mn(II), we used NMR to measure the [^15^N, ^1^H]-TROSY-HSQC spectra of ^15^N- and deuterated T7 primase in the presence of the selected metal ion (Fig. 4) and under the same conditions as for the biochemical experiments. Since Mn(II) is paramagnetic, it enhances the relaxation rate. The relaxation rate is indirectly proportional to the width of the signal; thus, enhanced relaxation rates lead to a broadening of the signal of spins close to the Mn(II) binding site. In addition, changes in the chemical environment of amino acids located further from the Mn(II) binding site can lead to chemical shift perturbations in a HSQC spectrum. After the addition of MgCl_2_ to T7 DNA primase, we observed significant chemical shift perturbations compared to those obtained for the metal-free sample of T7 DNA primase (Fig. 4a). Likewise, distinct chemical shift perturbations were observed for the Mn(II)-bound T7 primase.

To identify the amino acid residues situated in the proximity of the active site that mediate divalent metal ion binding, we assigned backbone resonances using the TROSY version of the triple resonance experiment. We measured the spectra of the T7 primase domain in three states: free, bound to magnesium, or bound to manganese. With the exception of the zinc-binding domain, 70% of the chemical shifts of the T7 primase have been assigned to their corresponding residues^40^. An expanded view of the chemical shift perturbations in the ^1^H-^15^N resonances of the indicated amino acids is presented in Fig. 4b and Table S1. The values of all the chemical shift perturbations that occurred in response to metal ion substitutions are presented in Fig. 4c. Complexation-induced changes in chemical shifts were mapped for the T7 primase bound to magnesium, or bound to manganese (Fig. 4d). Changes in chemical shifts observed in the ZBD of T7 primase are presumably due to a conformational change T7 primase undergoes^20^.

### Generalization of the Mn(II) vs. Mg(II) binding sites in proteins

Analysis of the amino acid composition of the Mg(II) or Mn(II) binding site in proteins facilitates predictions with good probability of the likelihood that an unresolved metal binding site will bind Mg(II) or Mn(II). To predict the likelihood of Mg(II) or Mn(II) binding to T7 primase, we derived the linear discriminant analysis (LDA) statistical model. In addition to their predictive power, the LDA results enabled us to identify the most prominent features of the amino acid compositions of the Mg(II) and Mn(II) binding sites and how they differ, results that could also be used to explain the augmented catalytic activity of primase when Mn(II) is substituted for Mg(II). The data used in the analysis were obtained from the PDB database consisting of 1370 enzyme crystal structures, half of which are bound to Mg(II) while the other half are bound to Mn(II). For each crystal structure, 25 features were extracted from its PDB file, including a count of the amino acids located within a 10 Å radius of the site and some geometric features (e.g., average pairwise distance between two amino acids), see Tables S2 and S3. The LDA classifier was described via a 25-dimensional vector w* and a number *τ*. The vector w* represents a maximum of the function *J*(*w*) (Eq. 1.1). The model (i.e., w* and *τ*) was derived using the 1370 crystal structures mentioned above and computed according to Eq. 1.2 (*see* Methods).

**Figure 5:**
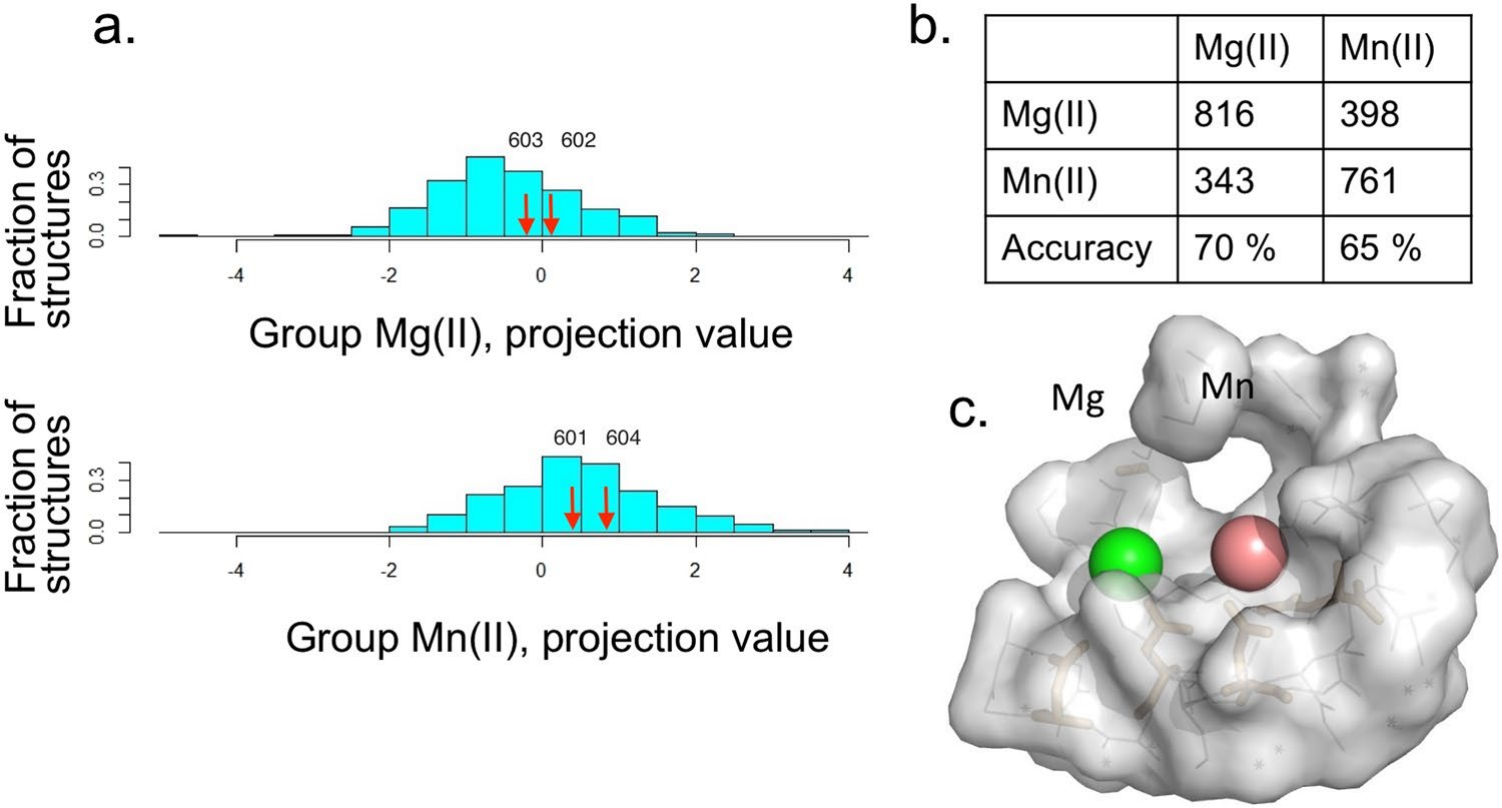
LDA analysis of the divalent metal binding site. (**a**) Histogram of projection values of 685 bound Mg(II) molecules (top) and 685 bound Mn(II) molecules (bottom) on the LDA vector w*. The separation between the two classes is evident: Mg(II) tends to have smaller (negative) projection values on w*, while Mn(II) tends to have larger (positive) projection values. Arrows schematically show the value of the projection of every feature vector on the LDA. Posterior probabilities are derived accordingly. (**b**) The confusion matrix for the LDA classifier using Leave-One-Out (LOO) cross-validation on the 2318 atoms. The columns represent the true classification and the rows are the LDA predictions. Accuracy (70% for Mg(II) and 65% for Mn(II)) should be compared against the baseline of a random guess (accuracy of 0.5). (**c**) Model of the active site of the T7 primase RNA polymerase domain containing Mg(II) and Mn(II) (based on the structure of the T7 primase domain of the gene 4 product, pdb code: 1 nui).

The binding site was predicted by calculating the projection of *b*, the 25-dimensional feature vector for that site, onto the LDA vector w* and then testing whether the projection satisfied 〈w*, *b*〉 ≤ *τ* (where 〈,〉 is the scalar inner product of two vectors). For projections that satisfied the relationship, Mg(II) was predicted; otherwise, Mn(II). In addition, posterior probabilities were assigned to the site according to the difference 〈w*, *b*〉 − *τ*. Figure 5 shows the distributions of the projections of the 1370 data points on w* for both metal ions. The shift to the right of the distribution of the Mn(II) projections relative to that of Mg(II) suggests that statistically significant separation is feasible. Indeed, as summarized in Table 1, the model correctly predicted 70% of the 685 Mg(II) sites and 68% of the 685 Mn(II) sites (prediction was done according to leave-one-out cross-validation; see Methods for description).

**Table 1:** Confusion matrix for the LDA classifier using Leave-One-Out (LOO) cross-validation on the 1370 atoms.

	Mg(II)	Mn(II)
Mg(II)	480	465
Mn(II)	205	220
Accuracy	70%	68%

The columns represent the true classification and the rows are the LDA predictions. The accuracy [70% for Mg(II) and 68% for Mn(II)] should be compared against the baseline of a random guess, the accuracy of which is 50%.

To interpret the LDA results, we examined the largest entries in absolute value in the vector w*. These entries correspond to the dominant features for distinguishing the binding site of Mg(II) from that of Mn(II). Two such features, the average distance to the binding site and the average pairwise distance as presented in Tables S2 and S3, pertain to the geometry of the metal binding site. In addition, an indicator of the likelihood to find various amino acid residues in close proximity to Mg(II) or Mn(II) may be derived from the LDA vector w* (Table S4).

Finally, we demonstrate how to apply the LDA model to the classification of an unknown metal binding site as Mn(II) or Mg(II) (see Methods). Four binding sites in the T7 DNA primase structure (from the homodimer assembly in the available crystal structure, PDB entry 1 nui) were re-examined using the LDA classification model. Table 2 presents the LDA prediction for metal binding according to the amino acid composition of the metallo-site and the posterior probabilities assigned by the LDA to each class. The LDA analysis indicated a binding preference for Mn(II) in one of metal binding site of the T7 DNA primase while its other metal biding site showed no preference for either metal (Table 2, Fig. 5). The LDA based analytical approach can be exploited for other, related systems.

**Table 2:** Results of machine-learning algorithm (LDA) application in the classification of enzyme active site features.

Atom	Posterior Mg(II)	Posterior Mn(II)	Classification
601	0.4	0.6	Mn(II)
602	0.47	0.53	Mg(II)/Mn(II)
603	0.53	0.47	Mg(II)/Mn(II)
604	0.32	0.69	Mn(II)

X-ray crystal structure data of T7 DNA primase (PDB entry 1 nui). Atoms designated as 601–604 were taken from the PDB file and represent the locations of the four divalent metal cofactors at the enzyme’s active site. Posterior probabilities were calculated according to the projection on the LDA vector of the feature vector of each atom.

## Discussion

Our study provides insight into the essential roles played by divalent metal cofactors in enzyme activity. Many enzymes, such as DNA and RNA polymerases and general ATPases, require Mg(II) for their catalytic activity. Kinetic assay studies have shown that the reaction catalyzed by an enzyme that utilizes Mg(II) can be initiated by adding Mg(II) to the reaction mixture. When Mn(II) is substituted for Mg(II), many enzymes, including general ATPases, DNA and RNA polymerases, and nucleases, among others, retain their activity, a fact that can be exploited for a variety of analytical needs^30^. However, whether enzyme activity is retained when the metal cofactor is exchanged depends on the concentrations of the divalent metal ions *in-vivo* and their affinities to the enzyme active site.

Present in the cell at millimolar concentrations^30^, Mg(II) is utilized mostly for electrostatic stabilization and electrophilic activation of the substrate. Indeed, it is the most common metal ion cofactor in enzymes, which is probably due to its ability to form stable complexes with phosphate-containing molecules such as ATP^29^. In cofactor substitution experiments with human primase, enzyme activity in the absence of Mg(II) was restored when it was supplied with Mn(II) and was augmented by the addition of Mn(II) to assays that contained 5 mM Mg(II)^35^, which is close to the concentration of Mn(II) *in vivo*. In addition, for some enzymes, like the DnaG of *Mycobacterium tuberculosis*, Mn(II) is the preferred cofactor for RNA primer synthesis^41^. In our study, after we substituted Mg(II) with Mn(II) in bacteriophage T7 DNA primase, enzyme primer synthesis activity was elevated. Titration of Mn(II) in assays lacking Mg(II) showed that while low concentrations of Mn(II) activated T7 DNA primase, high concentrations of the ion led to partial inhibition of the enzyme.

Among the metalloenzymes, some are monometallic such as general ATPases while others are bimetallic or even tri-metallic, such as polymerases^42^. Compared to the single-ion mechanism, the use of two metal ions has many advantages^26^, including (but not limited to) reduced activation barriers, better substrate accommodation, and easier electrostatic activation of the substrate. Furthermore, the formation of a low-energy transition state is predicted to be energetically favored in bimetallic enzymes, in which the presence of the second metal ion can reduce the strength of the bridging-ligand field potential and thereby enhance the enzyme’s binding affinity for other ligands, especially water (which can increase the Lewis acidity of the reaction)^26^. Recently, it was suggested that a third metal ion is involved in transition state stabilization^42^. Based on our computational analysis and the results of our NMR experiments, we hypothesize that at least one Mg(II) ion in the active site of T7 DNA primase can be replaced with a Mn(II) ion.

Functionally, the use of Mn(II) instead of Mg(II) increases NTP misincorporation by approximately three-fold on the synthetic DNA template d(ACC)_20_, and similarly minor effects on fidelity were observed for other DNA templates. The relatively modest effects that Mn(II) was found to have on fidelity suggest that it may be difficult to reduce the accuracy of an enzyme that under normal conditions is only marginally accurate. In addition to its slight effect on the rate of NTP misincorporation, Mn(II) can enhance the binding of noncognate NTPs to the primase-DNA complex.

The observations that Mn(II) stimulates primase activity and that the magnitude of stimulation is template-dependent elicits the prediction that Mn(II) alters the confined structure of the active site around the metal ion. Indeed, NMR measurements revealed structural changes in the Mn(II)-bound enzyme compared both to the free enzyme and to the Mg(II)-bound state. In addition, the identity of the divalent metal cofactor affects the enzymatic pathway of diribonucleotide synthesis by T7 primase. Specifically, when Mn(II) occupies the metal binding site, rapid diribonucleotide synthesis occurs, but release of these products from the enzyme active site is delayed. This pause in diribonucleotide release likely allows the primase domain to prevent dissociation of the primer synthesis intermediates from the enzyme’s active site before full-length primers are formed.

It is of note that the rate constant of dinucleotide release obtained in the presence of Mn(II) mirrors the *k*_cat_ that was recently observed for the formation of full-length primers^43^. This observation suggests that similar conformational changes are regulating the release of primer intermediates and full-length primers from the primase active site and that metal ligands may play a regulatory role in these protein motions by preventing the premature release of reaction intermediates.

The metal-dependent modulation of steps in the synthesis pathway may allow for the regulation of primer synthesis in the context of the replisome during the coordinated replication of the leading and lagging DNA strands. In addition, trans-acting regulators may exert an effect on the activity of T7 primase in response to environmental factors, as the concentration of free Mn(II) in *E. coli* cells increases in response to oxidative stress^44^. It is also possible that divalent metal cofactor identity regulates the response of T7 primase and other metal-dependent proteins to alarmone, polyamine, and polyphosphate pools.

Assessment of the binding propensity of different metals to an enzyme’s metal binding site by using LDA, a machine-learning classification algorithm, examines the structural properties of a small neighborhood of the site to predict binding preference. The model was applied to predict the binding preferences of four metal cofactor binding sites on the T7 DNA primase. While one site exhibited a binding preference for Mg(II), the other three seemed to have similar affinities for both Mg(II) and Mn(II). Likewise, the LDA method can be used to predict the binding propensities of Mg(II) and Mn(II) in the metal binding sites of other enzymes based on the amino acid composition of each enzyme’s active site. Thus, another conclusion that may be drawn from LDA model based evaluations of metal binding sites concerns the dominant structural features at the binding site that determine the binding propensities of Mg(II) and Mn(II). Knowledge of these structural variations can contribute to our general understanding of the divalent metal ion binding preferences of other enzymes.

## Methods

### Protein expression and purification

All chemical reagents were molecular biology grade (Sigma); ATP, CTP, (Roche Molecular Biochemicals); Radio-labeled ATP or CTP (800 Ci/mmol) were from Perkin Elmer. Molecular weight markers used in SDS-PAGE were Precision Plus Protein prestained standard (BioRad); Ready gel 10–20% linear gradient was purchased from BioRad (Hercules, CA); the T7 primase fragment (residue: 1–271) was over-produced and purified using metal free buffers as previously described^38^. For expression of isotopically enriched proteins, a starter culture was used to inoculate 2 L of M9 medium containing 50 µg/mL kanamycin and 1 g ^15^N-NH_4_Cl. The M9 was made in D_2_O for the expression of perdeuterated proteins. When the culture optical density (600 nm) reached approximately 0.8, it was induced for protein overexpression with 0.3 mM isopropyl-β-D-thiogalactopyranoside (IPTG) for 16 to 24 h at 16 °C. The cell pellet was resuspended in buffer A (50 mM Tris-HCl pH 7.5, 1 mM EDTA, 1 mM DTT), 100 mM NaCl, 0.25 mg/ml Lysozyme and 1 mM PMSF and incubated on ice for 1 h. After four freeze-thaw cycles, streptomycin sulfate was added until it reached a concentration of 1%. After centrifugation at 15,000 × g for 30 min, supernatant was diluted with buffer A (no salt) and loaded onto DEAE Sepharose (packed in AP-5, Waters, NJ) and washed with 200 mL of 50 mM Tris-HCl. The primase domain was eluted using a linear gradient of Buffer A from no salt to 1 M NaCl. Primase fractions were combined and ammonium sulfate was added (0.361 g/ml). The precipitate was dissolved in 10 mL buffer A and was loaded onto a superdex S-200HR column and was eluted using Buffer A. The fractions containing the primase domain were combined and applied to a 5-ml HiTrap Blue affinity column. The column was washed with excess amount of Buffer A and the primase was eluted using a linear gradient of NaCl (0–1 M) in Buffer A. The purified protein was dialyzed against Buffer B (50 mM KH_2_PO_4_/K_2_HPO_4_ pH 7, 1 mM DTT) and concentrated to 0.4 mM.

### Oligoribonucleotide synthesis assay

Synthesis of oligoribonucleotides by DNA primase was measured as described^38^ in reactions containing various concentrations (1.1, 3.3, and 10 µM) of the gene 4 primase fragment. A standard 10-µL reaction contained 5 µM of DNA template (5′-GGGTCA_10_-3′), 1 mM ATP, 1 mM CTP (containing 0.3 mCi/mL [α-^33^P] CTP), and primase fragments in a buffer containing 40 mM Tris-HCl pH 7.5, 8 mM MgCl_2_ or MnCl_2_, 10 mM DTT, and 50 mM potassium glutamate. After incubation at room temperature for 20 min, the reaction was terminated by adding an equal volume of sequencing buffer containing 98% formamide, 0.1% bromophenolblue, and 20 mM EDTA. The samples were loaded onto 25% polyacrylamide sequencing gel containing 7 M urea and visualized using autoradiography.

### Rapid-quench analysis of diribonucleotide synthesis

Multiple-turnover diribonucleotide synthesis reactions were assembled as follows: 10 µM T7 primase fragments and 100 µM DNA template with the primase recognition site 5′-GTCA_10_-3′ in reaction buffer (40 mM HEPES-KOH, pH 7.5, 50 mM K-glutamate, 5 mM DTT, 0.1 mM EDTA). The reaction was loaded in one syringe of a rapid quench-flow instrument (RQF-3, Kintek). The other syringe was loaded with a solution containing 1 mM ATP, 2 mM CTP (containing 0.3 mCi/mL [α-^32^P] CTP), and 10 mM MgCl_2_ or 2 MnCl_2_ in reaction buffer. The contents of the two syringes were mixed to start the reaction. All concentrations indicated are final concentrations after mixing and all assays were carried out at 25 °C. After the indicated incubation times have passed, the reaction was quenched with 125 mM EDTA, and 0.1% SDS followed by analysis by denaturing gel electrophoresis and phosphorimaging. The concentration of diribonucleotide product, determined by comparison of phosphorimage intensity to a standard dilution series of radiolabeled substrate, were normalized to enzyme concentration and plotted over time. For single-turnover diribonucleotide synthesis, 10 µM of T7 primase fragments, 100 µM GTCA_10_ template, and 1 nM [γ-^32^P] ATP in reaction buffer were loaded in one syringe of the RQF-3. The other syringe was loaded with a solution containing 2 mM CTP and 10 mM MgCl_2_ or 2 mM MnCl_2_ in reaction buffer. The reaction was started as above, quenched with 125 mM EDTA, and 0.1% SDS. Samples were analyzed by denaturing gel electrophoresis and phosphorimaging. The fraction of ATP converted into product was plotted as a function of time.

### Analysis of kinetics data

The time dependence of multiple-turnover diribonucleotide formation was fit to either a linear equation, [AC]/[PF] = *k*_cat_t, or to the pre-steady-state burst equation, [AC]/[PF] = A(1 - e^−*k*burstt^) + *k*_cat_t, where A is the amplitude of the pre-steady-state burst, *k*_burst_ is the observed rate constant for the pre-steady-state burst, *k*_cat_ is the steady-state rate constant for dinucleotide synthesis, and t is time, in seconds. The fraction of ATP converted to diribonucleotide in single-turnover reactions was fit to a single-exponential equation, fraction product = (1- e^−*k*obst^), where A is the reaction amplitude, *k*_obs_ is the observed rate constant for diribonucleotide formation, and t is time, in seconds. Data were fit to the indicated equations using KaleidaGraph (Synergy).

### NMR

[^1^H,^15^N]-TROSY HSQC spectra of ^15^N-deuterated T7 Primase in three states – free, bound to Mg(II), or bound to Mn(II) – were recorded at 25 °C on a Bruker DMX 800 MHz spectrometer equipped with TCI cryoprobes with Z gradient. Data were processed and analyzed using NMRPipe^45^ and NMRView^46^.

### The Statistical Method

Our data consist of 1370 molecules obtained from the PDB and divided into two classes of equal size according to the binding metal: Mg(II) or Mn(II). For each molecule, we computed a set of 25 attributes (features) as described in Table S2.

The statistical method we used, linear discriminant analysis (LDA), is best understood from a geometrical perspective (Fig. S1). With each molecule, we associated a 25-dimensional vector *x* that contains the values of the molecule’s attributes. Therefore, each molecule is simply a point in the 25-dimensional real plane $\,{{\mathbb{R}}}^{25}.$ As such, the 1370 molecules in the data set form a collection of points in the plane. Let us call *C*_*MG*_ and *C*_*MN*_ the sets of molecules with Mg(II) and Mn(II), respectively. Our statistical task was to find a classifier that separates *C*_*MG*_ from *C*_*MN*_. In what follows, we use the standard notation *x*,y for the inner product of two vectors $x=({x}_{1},{x}_{2},\ldots ,{x}_{p})\,{\rm{and}}\,y=({y}_{1},{y}_{2},\ldots ,{y}_{p}),{\rm{i}}{\rm{.e}}.,\langle x,y\rangle =\sum _{i=1}^{p}{x}_{i}\cdot {y}_{i}.$

LDA entails the search for a linear combination of variables (features) that best separates the data into two classes, i.e., identifying a set of coefficients *w* = (*w*_1_, *w*_2_, … *w*_*p*_) (in our case, *p* = 25), such that the two sets of numbers {*w*, *x*:*x* ∈ *C*_*MG*_} and {*w*, *x*:*x* ∈ *C*_*MN*_} are as distinct as possible (for example, one contains only positive numbers and the other only negative numbers).

The logic that drives the choice of *w* is as follows. Consider the samples plotted in Fig. S1. Two classes, *C*_*MG*_ and in *C*_*MN*_, are presented and marked in green and blue, respectively. The points are considered to lie in a *p*-dimensional (rather than a two-dimensional) space for an arbitrary number *p*. The consideration of another geometric fact, namely, that *w* is a unit vector (i.e., its Euclidean length is 1), renders the inner product 〈*w*, *x*〉 to be the size of the projection of *x* onto *w* (as demonstrated in Fig. 1), and its sign depends on the angle between the vectors.

Vector *w* is presented such that the projections of the points in *C*_*MG*_ and in *C*_*MN*_ on *w* are clearly separated. Fig. S1 shows two possible choices for *w*: one that provides good separation and one that does not. As such, the horizontal direction in Fig. S1 is preferable to its vertical counterpart because the former yields two desirable features: after the projection, the centers of the two classes (the black dots) are well separated, and the variance of each cluster is reduced. Simply put, the projection squeezes the clusters and pushes their centers apart.

We now turn to a mathematical description of LDA. The two objects that capture the notion of squeezing each cluster and separating the cluster centers are, respectively, the *within-class scatter matrix Sp* = *S*_1_ + *S*_2_, where *S*_1_ = $\sum _{j=1}^{n}({x}_{j}-{\mu }_{1}){({x}_{j}-{\mu }_{1})}^{T}$ is the scatter matrix of class 1 (*S*_2_ is defined similarly), and the *between-class scatter matrix S*_*B*_ = (*μ*_1_ − *μ*_2_)(*μ*_1 −_ *μ*_2_)^*T*^, where the vectors ${\mu }_{1},{\mu }_{2}\in {{\mathbb{R}}}^{25}$ are the class centers. We are looking for the vector w* that maximizes the following function *J*(*w*) (which is called the Fisher score):

11 $$J(w)=({w}^{T}{S}_{B}w)/({w}^{T}{S}_{P}w).$$

The nominator *w* ^*T*^ *S*_*B*_ *w* represents the distances between the projections of *μ*_1_ and *μ*_2_ onto *w* (which needs to be large), and the denominator *w* ^*T*^ *S*_*P*_ *w* represents the sum of variances of the two projected clusters (which needs to be small). The matrices *S*_*B*_ and *S*_*P*_ were computed using the attributes of the 1370 divalent metal binding sites (selected from the PDB). It turns out that w* has a closed formula,

12 $${w}^{\ast }={S}_{P}^{-1}({\mu }_{1}-{\mu }_{2}).$$

w* was used to perform the classification in the following way: Given a new site that whose binding preference is to be classified as for either Mg(II) or Mn(II), we first computed its feature vector *x* (according to Table S2), then we computed 〈*x*, w*〉, the projection of *x* onto w*, and the site is classified as Mg(II) if 〈*x*, w*〉 is closer to 〈*μ*_*MG*_, w*〉 or as Mn(II) if 〈*x*, w*〉 is closer to 〈*μ*_*MN*_, w*〉.

The standard method to evaluate whether the vector w* indeed provides a good partition is to compute the confusion matrix for a *leave-one-out cross validation*. Specifically, for each sample point, compute the LDA model when this point is set aside (namely, compute w* using the remaining *n* − 1 points). Then use the LDA vector w* to predict the class of the point that was set aside and record the prediction result (correct or erroneous).

#### Derivation of the chemical conclusions from the results of the LDA

The LDA vector w* may be examined to determine which features play dominant roles in the separation of the two classes. Recall that the classification of a data point *x* is performed according to the value of the inner product 〈*x*, w*〉. If all features have the same scale, then an entry in w* of a small absolute value contributes less to 〈*x*, w*〉 than an entry of large absolute value. Therefore, large absolute value entries indicate important features. In addition, the sign of each entry indicates which class typically has larger values for that feature. Let us take a concrete example. In Table S4, two of the largest (absolute value) entries in w* are for Lys and His. His has a positive value, and as Mn(II) projections tend more to the positive than those of Mg(II) (see Fig. 5), we can conclude that the presence of His is more likely in a binding site for Mn(II). Likewise, the negative value of the entry for Lys indicates that it is more likely to be found in the vicinity of a site with a binding preference for Mg(II). The value for Ser is almost zero, and therefore, we can conclude that its distribution is similar in the vicinity of either Mn(II) or Mg(II) binding sites.

## Electronic supplementary material

Supplementary Information
