# Supplementary material for: Modulation of RNA primer formation by Mn(II)-substituted T7 DNA primase
Source: Sci Rep. 2017 Jul 19;7:5797. doi: 10.1038/s41598-017-05534-3 (PMC5517523; doi:10.1038/s41598-017-05534-3)
Supplement: Supplementary file 1 — Supplementary Information [file 41598_2017_5534_MOESM1_ESM.pdf]

Supplementary Information

Modulation of RNA primer formation by Mn(II)-substituted T7 DNA primase

Stefan Ilic[1], Sabine R. Akabayov[1], Roy Froimovici[2], Ron Meiry[2], Dan Vilenchik[2], Alfredo Hernandez[3], Haribabu Arthanari[3] & Barak Akabayov[1*]

[1] Department of Chemistry, Ben-Gurion University of the Negev, Beer-Sheva, 8410501 Israel

[2] Department of Communication Systems Engineering, Ben-Gurion University of the Negev, Beer-Sheva, 8410501, Israel

[3] Department of Biological Chemistry and Molecular Pharmacology, Harvard Medical School, 240 Longwood Ave., Boston, Massachusetts 02115, USA

[*]email: akabayov@bgu.ac.il

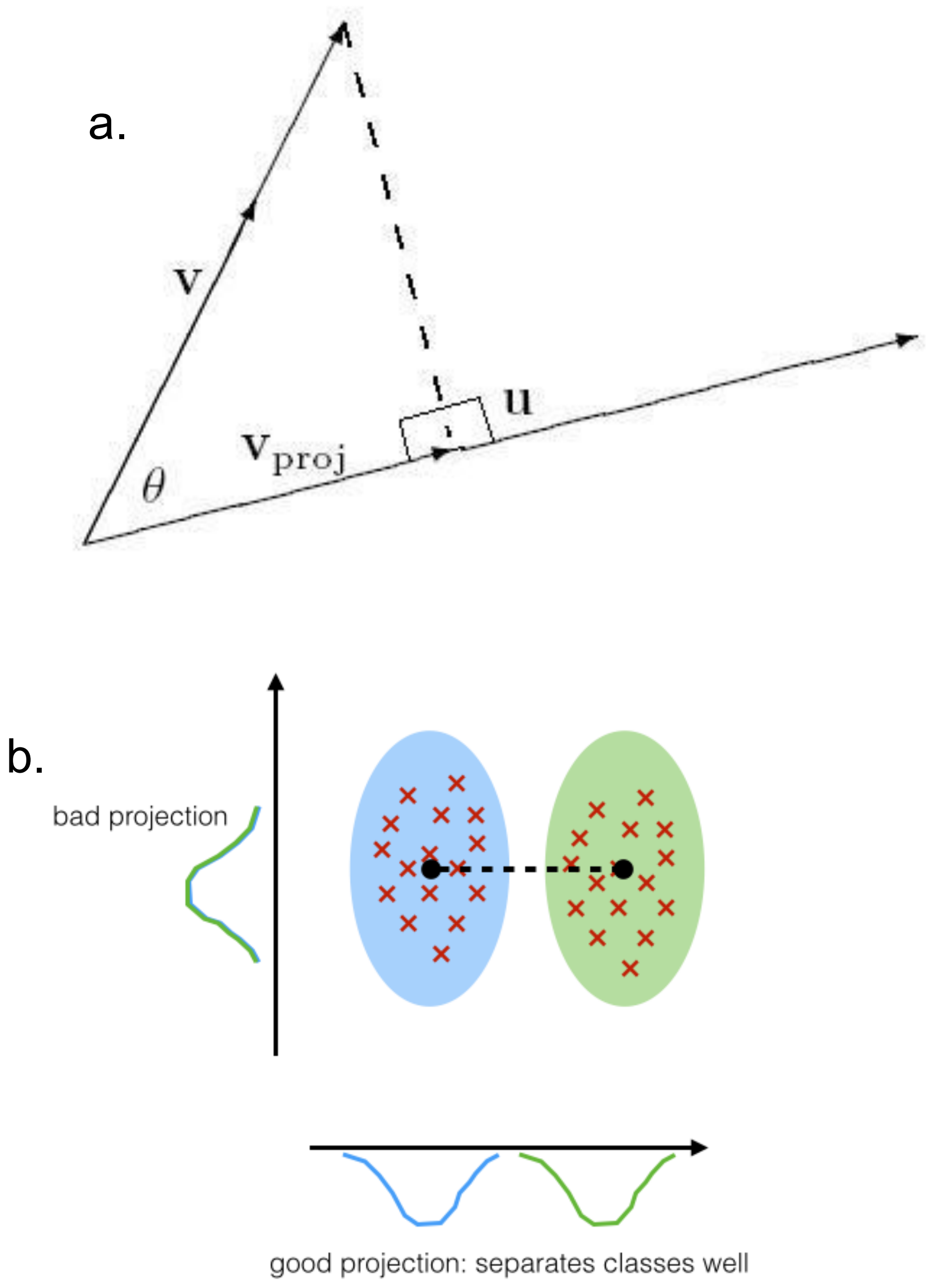

Figure S1 The LDA method. a) The projection of a vector v onto another vector u is given by v_proj=⟨u,⟩/‖ u‖ . b) Illustration of the LDA method on two-dimensional data. The LDA vector is chosen so that the distance between the two projected centers of mass is maximized while the variance of each class of points is minimized. In the example shown, the horizontal line is the correct choice

AA	Resid. #	Mg(II)	Mn(II)
VAL	10	0.91	2.33
PHE	11	0.98	1.8
LEU	12	1.08	2.6
TYR	13	0	0
HIS	14	0	0
ILE	15	0	0
PRO	16	0	0
CYS	17	1.12	0
ASP	18	0.87	1.48
ASN	19	0.99	1.07
CYS	20	1.18	0
GLY	21	1.01	0
SER	22	0.98	0
SER	23	1.04	0
ASP	24	0.92	0
GLY	25	1.04	0
ASN	26	0.99	0
SER	27	0.97	2.62
LEU	28	1.05	2.77
PHE	29	0.9	3.25
SER	30	0.9	2.59
ASP	31	0	0
GLY	32	1.19	2.84
HIS	33	0	0
THR	34	0.97	3.33
PHE	35	0	2.85
CYS	36	0.89	0
TYR	37	1.03	0
VAL	38	0	0
CYS	39	0	0
GLU	40	0	0
LYS	41	1.1	0
TRP	42	0	0
THR	43	0	2.6
ALA	44	1.04	2.17
THR	49	1.1	1.99
LYS	50	0	2.28
GLU	51	0.93	1.9
ARG	52	0	0
ALA	53	0.97	2.9
SER	54	1.1	2.65
LYS	55	1.28	0
ARG	56	0	0
LYS	57	0.92	2.6
PRO	58	0	0
SER	59	1.06	2.03
GLY	60	0	0
GLY	61	1.46	3.42
LYS	62	0	0
PRO	63	0	0
GLY	64	0	0
THR	65	0	0
TYR	66	0	0
ASN	67	0	0
VAL	68	0.92	3.02
TRP	69	1.13	0
ASN	70	1.15	2.67
PHE	71	1.35	3.62
GLY	72	1.14	3.53
GLU	73	1.06	3.31
SER	74	0.97	3.16
ASN	75	0.95	2.05
GLY	76	1.1	3.22
ARG	77	0	0
TYR	78	0	2.5
SER	79	1.17	0
ALA	80	1.07	0
LEU	81	1.15	0
THR	82	0	0
ALA	83	0	0
ARG	84	0	0
GLY	85	1.04	0
ILE	86	0	0
SER	87	1.27	NA
LYS	88	1	1.28
GLU	89	0.92	0
THR	90	0	0
CYS	91	0	0
GLN	92	0	0
LYS	93	0	0
ALA	94	0	0
GLY	95	1.06	0
TYR	96	1.08	0
TRP	97	1.18	0
ILE	98	1.05	0
ALA	99	1.12	0
LYS	100	0.85	2.24
VAL	101	0.97	2.71
ASP	102	1.01	0
GLY	103	0.9	2.33
VAL	104	0.79	2.38
MET	105	1.04	1.86
TYR	106	1.02	0
GLN	107	0	0
VAL	108	0.79	NA
ALA	109	1.58	0.96
ASP	110	1.16	0
TYR	111	1.23	NA
ARG	112	1.2	2.95
ASP	113	0	2.86
GLN	114	1	2.52
ASN	115	0	0
GLY	116	1.02	3.06
ASN	117	0	2.62
ILE	118	0	0
VAL	119	0	0
SER	120	0	0
GLN	121	0	0
LYS	122	0	0
VAL	123	0	0
ARG	124	0	0
ASP	125	0	0
LYS	126	0	0
ASP	127	0	0
LYS	128	0	0
ASN	129	0	0
PHE	130	0	0
LYS	131	0	0
THR	132	0	0
THR	133	0	0
GLY	134	0	0
SER	135	1.12	2.58
HIS	136	1.47	0
LYS	137	0	0
SER	138	0	0
ASP	139	0	0
ALA	140	0	0
LEU	141	0	0
PHE	142	1.1	NA
GLY	143	1.04	0
LYS	144	0.87	0
HIS	145	0.9	0
LEU	146	0	0
TRP	147	0	0
ASN	148	0	0
GLY	149	0.9	2.44
GLY	150	0.99	2.48
LYS	151	0	0
LYS	152	0	0
ILE	153	0	0
VAL	154	0	0
VAL	155	0	0
THR	156	0	0
GLU	157	0	0
GLY	158	0	0
GLU	159	0	0
ILE	160	0	0
ASP	161	0	0
MET	162	0	0
LEU	163	0	0
THR	164	0	0
VAL	165	0	0
MET	166	0	0
GLU	167	0	0
LEU	168	0	0
GLN	169	0	0
ASP	170	0	0
CYS	171	0	0
LYS	172	0	0
TYR	173	0	0
PRO	174	0	0
VAL	175	0	0
VAL	176	0	0
SER	177	0	0
LEU	178	0	0
GLY	179	0	0
HIS	180	0	0
GLY	181	0	0
ALA	182	0	0
SER	183	0	0
ALA	184	0	0
ALA	185	0	0
LYS	186	0	0
LYS	187	0	0
THR	188	0	0
CYS	189	0	0
ALA	190	0	0
ALA	191	0	0
ASN	192	0	0
TYR	193	0	0
GLU	194	0	0
TYR	195	0	0
PHE	196	0	0
ASP	197	0	0
GLN	198	0	0
PHE	199	0	0
GLU	200	0	0
GLN	201	0	0
ILE	202	0	0
ILE	203	0	0
LEU	204	0	0
MET	205	0	0
PHE	206	0	0
ASP	207	0	0
MET	208	0	0
ASP	209	0	0
GLU	210	0	0
ALA	211	0	0
GLY	212	0	0
ARG	213	0	0
LYS	214	0	0
ALA	215	0	0
VAL	216	0	0
GLU	217	0	0
GLU	218	0	0
ALA	219	0	0
ALA	220	0	0
GLN	221	0	0
VAL	222	0	0
LEU	223	0	0
PRO	224	0	0
ALA	225	1.06	2.28
GLY	226	1.04	2.55
LYS	227	0.89	2.23
VAL	228	0	1.54
ARG	229	0.95	NA
VAL	230	0	0
ALA	231	0.97	NA
VAL	232	1.01	NA
LEU	233	0	0
PRO	234	0	0
CYS	235	0	0
LYS	236	0	0
ASP	237	1.6	NA
ALA	238	0	0
ASN	239	0	0
GLU	240	0	0
CYS	241	0	0
HIS	242	0	0
LEU	243	0	0
ASN	244	0	0
GLY	245	0	0
HIS	246	0	0
ASP	247	0	0
ARG	248	0	0
GLU	249	0	0
ILE	250	0	0
MET	251	0	0
GLU	252	0	0
GLN	253	0	0
VAL	254	0	0
TRP	255	0	0

Table S1 Summary of NMR results, peak assignments and their shifts upon metal addition. The chemical shift perturbations as calculated using $((\Delta H)^2 + (\Delta N/5)^2)^{0.5}$ where the difference in the ^{15}N and ^{1}H chemical shifts for samples with Mg^{2+} and Mn^{2+} were calculated with reference to the apo protein without the metal ions. NA denotes peaks that where broadened, hence chemical shift perturbations cannot be determined.

Three-letter code for amino acid	Number of the indicated amino acids within a distance of 10Å from the binding site
AVG DIST	Average distance to the binding site
DIST VAR	Variance of the distance to the binding site
AVG PAIRWISE DIST	Average pairwise distance between all pairs of amino acids
PAIRWISE DIST VAR	Variance of the pairwise distance
#Atoms	Total number of atoms within a distance of 10Ǻ of the site

Table S2 Feature set used in the paper for the LDA analysis. The features are of two types: the first 20 correspond to the number of each of the 20 amino acids situated within a distance of 10Å from the binding site. The next four features pertain to the topology around the binding site: the average distance between two amino acids (and the variance), and the average distance between an amino acid and the binding site (and variance). The last feature is the total number of atoms within a distance of 10Å from the binding site.

AVG DIST	DIST VAR	AVG PAIRWISE DIST	PAIRWISE DIST VAR	#Atoms
0.47	-0.08	0.23	0.001	-0.02

Table S3 Two prominent geometric features of the amino acid arrangement around the binding site contribute to the separation between Mn(II) and Mg(II). The first is the average distance from an amino acid to the binding site (averaged over all amino acids at distances equal to or smaller than 10 Å from the site). The second feature is the average pairwise distance for all pairs of amino acids at a distance of 10 Å from the binding site. The finding that the signs of both features are positive is indicative of the tendency toward larger metal binding sites in molecules with a binding preference for Mn(II).

TYR	ASN	GLN	ARG	LYS	HIS	ASP	GLU
-0.02	0.1	-0.17	-0.09	-0.36	0.45	0.21	0.08

GLY	ALA	VAL	LEU	ILE	PHE	PRO	TRP	MET	SER	THR	CYS
-0.15	-0.05	-0. 12	-0.16	0.07	0. 12	0.25	0.37	0.003	0.008	-0.08	0. 1

Table S4 Highlighted amino acids indicate those that the LDA model predicts are making the most significant contributions to the separation between Mn(II) and Mg(II) binding sites (Lys, His, and Trp are with the highest contribution). A positive or negative value indicates that the amino acid is more likely to be present in a Mn(II) or a Mg(II) binding site, respectively.
